# Neck circumference associated with arterial blood pressures and hypertension: A cross-sectional community-based study in northern Han Chinese

**DOI:** 10.1038/s41598-017-02879-7

**Published:** 2017-06-01

**Authors:** Shujun Fan, Boyi Yang, Xueyuan Zhi, Jing He, Ping Ma, Luyang Yu, Quanmei Zheng, Guifan Sun

**Affiliations:** 1 Research Center of Environment and Non-Communicable Disease, School of Public Health, China Medical University, Shenyang, 110122 China; 2 Guangzhou Key Laboratory of Environmental Pollution and Health Risk Assessment, Department of Preventive Medicine, School of Public Health, Sun Yat-sen University, Guangzhou, China; 3 Department of Non-Communicable Disease, Shenhe Center for Disease Control and Prevention, Shenyang, Liaoning China

## Abstract

Although several studies have investigated the associations of neck circumference (NC) with arterial blood pressures (BPs) and hypertension, no such studies have been conducted among Northern Chinese population. Between April and June 2015, a total of 2631 subjects aged ≥35 years old were recruited from Northeastern China. NC and arterial BPs were measured by trained personnel. Generalized linear and logistic regression analyses were applied to examine the associations of NC with arterial BPs and hypertension risk. The optimal cut-off points of NC for predicting hypertension were assessed by the receiver operating characteristic analysis. We found that NC was significantly associated with arterial BPs and hypertension risk in the Northeastern Chinese adults, even after adjusting for many covariates including body mass index, waist circumference or waist-to-hip ratio. The optimal cut-off values for NC to predict hypertension differed with sex, age, and body mass index. Our study suggests that NC may play an independent role in predicting hypertension beyond the classical anthropometric indices, and that it could be used as a valuable anthropometric measurement for routine assessment in primary care clinics and future epidemiological studies.

## Introduction

Hypertension is a leading risk factor for many disorders including coronary heart disease and stroke, and contributes to approximately 12.8% of annual deaths worldwide^1^. The causes of hypertension are complex and are correlated with numerous environmental and genetic factors. Among them, adiposity is a well-documented modifiable risk factor. Body mass index (BMI), waist circumference (WC) and waist-to-hip (WHR) are the most used anthropometric indices to reflect the total body adiposity or visceral adiposity, and to predict hypertension risk^2–4^. However, several recent studies reported that the regional deposition of fat, especially in the upper body segment, is more pathogenic than total body adiposity and visceral abdominal fat^5, 6^.

Neck circumference (NC), a simple, cheap, time-saving and practical anthropometric parameter, has been used as a surrogate measure for the upper body subcutaneous adipose tissue distribution. In the past decade, accumulating evidence showed that NC was independently associated with metabolic syndrome^7, 8^, obstructive sleep apnea syndrome^8^, and cardiovascular diseases^9–14^. However, only a few studies have investigated the relationships of NC with arterial blood pressures (BPs) and hypertension^9, 12, 14^, and the results were often inconsistent. In a systematic literature review, we found 3 human epidemiological studies on NC and arterial BPs or hypertension in Chinese population. Specifically, Zhou *et al*. investigated the relationships of NC with cardio-metabolic syndrome risk in 4201 middle-aged Chinese southerners, and found that NC had significant associations with arterial BPs and hypertension^12^. Similarly, a more recent study conducted in 1943 Central Chinese reported that the positive predictive of NC for discriminating hypertension were 77.58% in males and 71.36% in females^13^. However, another study on 1709 adults from central China, showed that NC was not associated with systolic blood pressure (SBP) and the association with diastolic blood pressure (DBP) was very weak^14^.

In China, the distribution of hypertension and obesity prevalence rates show a large geographical imbalance and people living in north areas usually have higher prevalence of the two disorders than the southerners, presumably due to differences in environmental exposure, dietary patterns, life habits, and genetic backgrounds^15–17^. However, the 3 prior studies mentioned above were all performed in Southern and Central China, and, to the best of our knowledge, no such study had been performed in people living in northern China. In addition, several prior studies conducted in other countries have applied receiver operating characteristic (ROC) analysis to assess the accuracy of NC as diagnostic tests for hypertension^18–20^ and indicated that NC may be a valuable anthropometric parameter to predict hypertension risk. However, no study to date has explored the optimal cut-offs of NC in discriminating hypertension in Chinese adults.

Therefore, we conducted a large community-based cross-sectional study in Northeastern China to explore (1) the potential relationships of NC with arterial BPs and hypertension; (2) the optimal cut-points of NC for predicting hypertension risk.

## Results

### Participant characteristics

**Figure 1:**
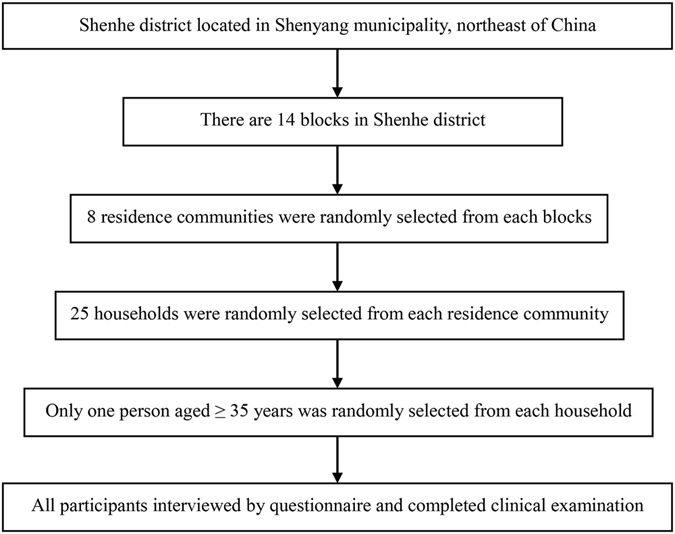
The sampling procedure of the study.

Figure 1 shows the sampling procedure of this study. A total of 2631 participants were included with a mean age of 59.78 ± 10.66 years. The demographic, clinical, lifestyle and anthropometric characteristics of the survey participants, overall and stratified by gender, are summarized in Table 1. The average NC was 35.40 ± 4.41 cm, and the overall prevalence of hypertension was 42.38%. Compared with females, males manifested significantly higher NC, BMI, WC, hip circumference (HC), WHR, SBP, DBP, triglycerides (TG), and lower total cholesterol (TC), low-density lipoprotein (LDL-C) and high-density lipoprotein (HDL-C) levels, and were more likely to be smokers, drinkers, and have higher education levels (all *p* < 0.05).

**Table 1:** Baseline characteristics of the study subjects by gender.

Variables	Total (n = 2631)	Females (n = 1756)	Males (n = 875)	*p*
Age groups, n (%)				0.010
35~44 years	260 (9.88)	183 (10.42)	77 (8.80)	
45~64 years	1491 (56.67)	959 (54.61)	532 (60.80)	
≥64 years	880 (33.45)	614 (34.97)	266 (30.40)	
Neck circumference (cm)	35.40 ± 4.41	34.30 ± 3.95	37.62 ± 4.46	<0.001
BMI (kg/m^2^)	24.46 ± 3.68	24.28 ± 3.53	24.83 ± 3.93	<0.001
Waist circumference (cm)	85.29 ± 9.14	83.19 ± 8.56	89.50 ± 8.80	<0.001
Hip circumference (cm)	97.40 ± 9.08	96.19 ± 9.03	99.83 ± 8.70	<0.001
Waist-to-hip ratio	0.88 ± 0.07	0.87 ± 0.07	0.90 ± 0.06	<0.001
Systolic blood pressure (mmHg)	129.70 ± 13.78	128.46 ± 14.27	132.19 ± 12.39	<0.001
Diastolic blood pressure (mmHg)	79.61 ± 9.06	78.49 ± 8.64	81.85 ± 9.45	<0.001
Fasting blood glucose (mmol/L)	5.80 ± 2.62	5.75 ± 2.67	5.92 ± 2.51	0.107
Triglycerides ^*^ (mmol/L)	1.59 (1.12, 2.07)	1.55 (1.12, 2.00)	1.63 (1.14, 2.25)	0.001
Total cholesterol (mmol/L)	5.20 ± 1.13	5.29 ± 1.17	5.03 ± 1.02	<0.001
Low-density lipoprotein (mmol/L)	2.74 ± 0.86	2.79 ± 0.88	2.64 ± 0.82	<0.001
High-density lipoprotein (mmol/L)	1.48 ± 0.49	1.52 ± 0.51	1.38 ± 0.41	<0.001
Salt consumption (grams/day)	6.67 (5.33, 9.33)	6.67 (5.33, 9.33)	6.67 (5.33, 9.33)	0.510
Family history of hypertension, n (%)	835 (31.74)	541 (30.81)	294 (33.60)	0.155
Education level, n (%)				0.008
No formal education	46 (1.75)	36 (2.05)	10 (1.14)	
Primary or secondary	1120 (42.57)	772 (43.96)	348 (39.77)	
High or technical secondary	852 (32.38)	569 (32.40)	283 (32.34)	
College or higher	613 (23.30)	379 (21.58)	234 (26.74)	
Smoking status, n (%)				<0.001
None-smokers	2193 (83.35)	1688 (96.13)	505 (57.71)	
Former-smokers	82 (3.12)	10 (0.57)	72 (8.23)	
Current–smokers	356 (13.53)	58 (3.30)	298 (34.06)	
Drinking status, n (%)				<0.001
None-drinkers	1904 (72.37)	1505 (85.71)	399 (45.60)	
Former-drinkers	45 (1.71)	14 (0.80)	31 (3.54)	
Current–drinkers	682 (25.92)	237 (13.50)	445 (50.86)	
Physical activity, n (%)				0.080
High level	1830 (69.56)	1241 (70.67)	589 (67.31)	
Low level	801 (30.44)	515 (29.33)	286 (32.69)	

*p* - value < 0.05 was considered statistically significant.

### Relationships of NC with arterial BPs

Firstly, we performed univariate linear regression analysis to explore the associations of the conventional predisposing factors with arterial BPs. As shown in Table S1, the *p* values for age, gender, fasting blood glucose (FBG), salt consumption, family history of hypertension, smoking and drinking status, physical activity and history of hypertension were less than 0.2. Thus, these variables were included in the final multivariate regression model. Further, we made additional adjustments for BMI, WC and WHR one by one due to close collinearity between them. For all subjects, NC was significantly associated with increased SBP and DBP levels in all the 5 models. For females, after further adjustments for BMI (model 3) or WC (model 4), the effect sizes were attenuated but still significant. For males, NC was associated with SBP in all the 5 models. However, for DBP, its association with NC became insignificant after additional adjustments for BMI or WC (Table 2).

**Table 2:** Estimated absolute increases in blood pressure (mmHg) with 95% CI per unit of neck circumference.

	Total			Females			Males		
	Estimate	95% CI	*p*_*1*_	Estimate	95% CI	*p*_*2*_	Estimate	95% CI	*p*_*3*_
SBP									
Model 1	0.63	0.51–0.75	<0.001	0.59	0.43–0.76	<0.001	0.50	0.32–0.68	<0.001
Model 2	0.34	0.22–0.45	<0.001	0.37	0.22–0.52	<0.001	0.28	0.11–0.45	0.001
Model 3	0.25	0.14–0.37	<0.001	0.26	0.10–0.41	0.001	0.26	0.08–0.43	0.004
Model 4	0.22	0.10–0.33	<0.001	0.22	0.06–0.37	0.006	0.24	0.06–0.42	0.008
Model 5	0.32	0.20–0.43	<0.001	0.35	0.20–0.50	<0.001	0.27	0.10–0.44	0.002
DBP									
Model 1	0.41	0.33–0.49	<0.001	0.34	0.24–0.45	<0.001	0.30	0.16–0.44	<0.001
Model 2	0.23	0.15–0.31	<0.001	0.25	0.15–0.35	<0.001	0.17	0.03–0.30	0.016
Model 3	0.17	0.09–0.25	<0.001	0.19	0.09–0.29	<0.001	0.12	−0.01–0.26	0.072
Model 4	0.16	0.08–0.24	<0.001	0.18	0.08–0.28	0.001	0.11	−0.03–0.25	0.121
Model 5	0.22	0.14–0.30	<0.001	0.25	0.15–0.35	<0.001	0.16	0.03–0.30	0.019

SBP, systolic blood pressure; DBP, diastolic blood pressure; CI, confidence interval.

Model 1, unadjusted;

Model 2, adjusted for age, fasting blood glucose, smoking and drinking status, salt consumption, physical activity, family history of hypertension, and history of hypertension;

Model 3, adjusted for model 2 + body mass index categories;

Model 4, adjusted for model 2 + waist circumference categories;

Model 5, adjusted for model 2 + waist-to-hip ratio.

### Relationship of NC with hypertension risk

We also performed univariate and multivariate logistic regression models to estimate the relationships of NC levels with hypertension risk (Table S2 and Table 3), and found that NC was significantly associated with hypertension risk in all the 5 models. Stratified analyses by gender also revealed a significant association in both males and females (All *p* < 0.05).

**Table 3:** Association between neck circumference (continuous data) and the risk of hypertension.

	Total			Females			Males		
	OR	95% CI	*p*_*1*_	OR	95% CI	*p*_*2*_	OR	95% CI	*p*_*3*_
Model 1	1.07	1.05–1.09	<0.001	1.06	1.03–1.09	<0.001	1.07	1.04–1.11	<0.001
Model 2	1.06	1.04–1.09	<0.001	1.06	1.03–1.09	<0.001	1.07	1.03–1.10	<0.001
Model 3	1.04	1.02–1.06	<0.001	1.03	1.01–1.06	0.014	1.05	1.02–1.09	0.002
Model 4	1.05	1.03–1.07	<0.001	1.04	1.01–1.07	0.006	1.06	1.02–1.10	0.001
Model 5	1.06	1.04–1.08	<0.001	1.06	1.03–1.08	<0.001	1.07	1.03–1.10	<0.001

OR, odds ratio; CI, confidence interval.

Model 1, unadjusted;

Model 2, adjusted for age, fasting blood glucose, smoking and drinking status, salt consumption, physical activity and family history of hypertension;

Model 3, adjusted for model 2 + body mass index categories;

Model 4, adjusted for model 2 + waist circumference categories;

Model 5, adjusted for model 2 + waist-to-hip ratio.

Subsequently, we used the areas under the ROC curves (AUCs) to evaluate the predictive values of NC for hypertension susceptibility (Table 4). The AUCs of NC were 0.577 and 0.583 for the risk of hypertension in females and males, respectively. NC of 32.75 cm for females and 35.75 cm for males were optimal cut-off points of combined sensitivity and specificity in identifying hypertension. Considering there are different NC levels in various age and BMI groups, we also assessed the optimal cut-offs of NC for hypertension by age groups and BMI categories. For age groups, ROC analysis showed that the AUCs for NC and hypertension was highest in 35–44 age group (0.619), and followed by 45–64 (0.584) and ≥65 age groups (0.573). The corresponding best cut-offs for determining hypertension was 38.50 cm, 32.25 cm and 34.25 cm, respectively. For BMI categories, the ROC cut-off values were observed at 31.55 cm (AUC: 0.559) for normal weight, 32.40 cm (AUC: 0.551) for overweight, and 33.30 cm (AUC: 33.30) for obesity.

**Table 4:** Gender, age and BMI-specific areas under the receiver operating characteristic curves showing the ability of neck circumference to identify subjects with hypertension.

	AUC	95% CI	Cut point (cm)
Gender			
Females	0.577	0.550–0.604	32.75
Males	0.583	0.546–0.621	35.75
Age (years)			
35–44	0.619	0.542–0.695	38.50
45–64	0.584	0.555–0.613	32.25
≥65	0.573	0.536–0.611	34.25
BMI (kg/m^2^)			
Normal	0.559	0.526–0.593	31.55
Overweight	0.551	0.517–0.585	32.40
Obese	0.584	0.515–0.652	33.30

BMI, body mass index; AUC, areas under the receiver operating characteristic curves; CI, confidence interval.

Furthermore, we assessed the associations between NC categories based on cut-off values and the risk of hypertension by gender (Table 5). Therefore, NC < 32.75 cm and <35.75 cm were the references in females and males, respectively. The results showed that in models fully adjusted for all the covariates and BMI, WC or WHR (models 3, 4 and 5), NC was significantly correlated with hypertension risk in both females and males.

**Table 5:** Association between neck circumference (categorical data) and the risk of hypertension.

	Total			Females^*^			Males^*^		
	OR	95% CI	*p*_*1*_	OR	95% CI	*p*_*2*_	OR	95% CI	*p*_*3*_
Model 1	1.83	1.54–2.18	<0.001	1.82	1.47–2.26	<0.001	1.82	1.34–2.46	<0.001
Model 2	1.76	1.47–2.11	<0.001	1.79	1.43–2.24	<0.001	1.71	1.25–2.34	0.001
Model 3	1.53	1.27–1.84	<0.001	1.51	1.20–1.90	<0.001	1.57	1.14–2.17	0.005
Model 4	1.54	1.28–1.87	<0.001	1.54	1.22–1.94	<0.001	1.59	1.15–2.21	0.006
Model 5	1.72	1.43–2.06	<0.001	1.74	1.39–2.17	<0.001	1.71	1.25–2.36	0.001

^*^for females, neck circumference < 32.75 cm was the reference; for males, neck circumference < 35.75 cm was the reference.

OR, odds ratio; CI, confidence interval.

Model 1, unadjusted;

Model 2, adjusted for age, fasting blood glucose, smoking and drinking status, salt consumption, physical activity and family history of hypertension;

Model 3, adjusted for model 2 + body mass index categories;

Model 4, adjusted for model 2 + waist circumference categories;

Model 5, adjusted for model 2 + waist-to-hip ratio.

## Discussion

In this study, we found that NC was associated with arterial BPs and hypertension risk in the Northeast urban Chinese adults, even after adjusting for many covariates including BMI, WC or WHR. In addition, we estimated the optimal cut-off value for NC to predict hypertension, and found that the values differed with gender, age and BMI.

### NC with arterial BPs and hypertension

In recent years, a growing number of studies have been performed to estimate the associations of NC with arterial BPs and hypertension. For instance, in a longitudinal cohort study of 431 Israeli individuals, Ben-Noun *et al*. reported that NC was strongly associated with SBP and DBP levels^21^. In a cross-sectional study of 4201 Chinese adults, Zhou and colleagues found that NC had positive correlation with SBP, DBP and hypertension^12^. Additionally, Lee *et al*. compared the predictive power of anthropometric indices for hypertension in a Korean population, and found that NC was a valuable index in distinguishing hypertension and normotension^19^. Consistent with these prior findings, our current study found that NC was significantly associated with both arterial BPs and hypertension.

Due to the close link of NC with BMI and WC, several previous studies found that after further adjustments of BMI or WC, the associations of NC with arterial BPs and hypertension became insignificant^9, 18^. For example, in the Framingham Heart Study in 3307 participants, Preis *et al*. found that NC were related with SBP and DBP levels and hypertension risk in both males and females after adjusting for several potential covariates. However, after further adjustments for BMI, NC was positively related to DBP in males only^9^. Likewise, in a cross-sectional study of Caucasion population, Assyov *et al*. observed that NC was associated with hypertension in univariate analysis. However, when adjusted for WC and age, the association lost its statistical significance in females^18^. Contrary with these studies, our study showed that the significance of the associations of NC with arterial BPs and hypertension were not changed after additional adjustments for BMI or WC, although the magnitudes of the associations were slightly attenuated. The inconsistencies between those prior studies and ours may be caused by differences in genetic backgrounds, dietary habits, adjustment factors, and the effects of other confounding factors.

### Optimal cut-off points of NC on hypertension risk

We further used the ROC analysis to determine the predictive validity of NC and evaluated optimal cut-off values for identifying hypertension, and found that a NC of more than 32.75 cm in females and 35.75 cm in males is suitable for assessing the likelihood of hypertension. In a systematic MEDLINE search, we found 4 relevant human epidemiological studies on the accuracy of NC as diagnostic test for hypertension. A study of 255 Caucasian adults found that the cut-off values of NC for the prediction of hypertension in females and males were 35 cm and 38 cm, respectively, which were relatively greater than those observed in our analysis^18^. Additionally, Zhou *et al*. reported that NC was a valuable index for discriminating hypertension in Chinese adults (AUCs were 0.635 and 0.659 for males and females, respectively)^12^, which further corroborated by the subsequent studies among Korean population (AUCs were 0.617 and 0.645 for males and females respectively)^19^ and American children (AUCs were 0.75 and 0.72 for boys and girls, respectively)^20^. In our study, the AUCs for males and females were 0.583 (95% CI = 0.546–0.621) and 0.577 (95% CI = 0.550–0.604), respectively, which were relatively lower than those observed in the previous studies. However, in the current study we observed that both the discriminative values in distinguishing hypertension were statistically significant (All *p* values < 0.001). Additionally, applying binary logistic regression analyses, we explored the association of NC with hypertension risk based on the calculated cut-off values of ROC analysis, and observed that participants with NC above cut-off values had an approximately 1.70-fold increased risk of hypertension than those with NC below cut-off values. In sum, although there exists heterogeneity in the diagnostic value of NC in discriminating hypertension among different populations, evidence from our and those previous studies consistently support that NC is a valuable index for discriminating hypertension.

Because of the links of age and BMI with NC, we further assessed the optima cut-offs of NC by age and BMI groups, and found that the cut-off values were relatively higher in the younger and those with higher BMI. To our knowledge, this is the first study to determine the age- and BMI-specific predictive validity of NC in identifying hypertension, thus it is difficult to directly compare our results with other findings. Future studies with large sample size and longitudinal design is needed to validate our findings.

### Potential mechanisms

The precise mechanisms underlying the relationships of NC with arterial BPs and hypertension are not fully understood. Several investigators have proposed that upper-body subcutaneous fat might affect the arterial BPs and the development of hypertension via releasing large amounts of systemic free fatty acid, which may induce insulin resistance, vascular injury, provoke endothelia cell dysfunction, and increase oxidative stress and very-low-density lipoprotein cholesterol production^22–26^. Additionally, NC is an important predictor of obstructive sleep apnea syndrome. Obstructive sleep apnea syndrome can cause hypertension by increasing sympathetic activity, which influences vascular resistance and cardiac output^27^, as well as the potential for fluid retention^28, 29^. Thus, the association of NC with arterial BPs and hypertension may also be mediated by its relationship with the severity of sleep-disordered breathing.

### Limitations and strengths

In interpreting the findings of the current study, three main limitations should be acknowledged. Firstly, the cross-sectional nature of the present study may limit the interpretation of causality of associations between NC and arterial BPs and hypertension. Secondly, self-reported information such as smoking and drinking status, and physical activity might have caused recall biases. In addition, we used questionnaire method to evaluate salt intake, which might have caused underestimation of actual salt intake^30–32^. Thirdly, NC is a proxy for upper-body subcutaneous fat, we did not perform radiographic measures to directly quantify the depot of fat. Despite these caveats, our study still has several advantages. First, to the best of our knowledge, it explored the associations of NC with arterial BPs and hypertension risk for the first time in the Northern Chinese adults, and reported the optimal sex-, age-, and BMI-specific NC cut-off points in the population. Second, all participants were of Han nationality and from the same district, which reduce the selective bias and improve the validity of statistical analysis.

## Conclusion

In conclusion, this is the first study to explore the relationship between NC and BPs in Northern Chinese. Our findings suggest that NC may play an independent role in predicting hypertension beyond the classical anthropometric indices including WC, BMI, and WHR, and that the optimal cut-offs of NC for distinguishing hypertension differs with sex, age and BMI. The measurement of NC is inexpensive and easier to obtain, thus it could be used as an important measure to consider for routine assessment in primary care clinics and other health care settings as well as for large-scale epidemiological studies on obesity-related diseases. However, considering the limitations of our study, further large prospective population-based studies and mechanistic studies are still needed to validate our finding, and to explain how NC contributes to the elevated arterial BPs and the development of hypertension.

## Methods

### Study participants and inclusion criteria

The cross-sectional study was conducted in Shenhe district between April and June 2015. The district locates in the center of Shenyang city and consists of 14 blocks. A total of over one million people reside in this district, of whom 73.00% are registered residents. A 3-stage sampling method was applied to select participants in this study. In the first stage, we selected all the 14 blocks of the district, and from each of these, 8 communities were randomly identified. In the second stage, we selected 25 households from each of the residence community using systematic sampling. In the third stage, one participant, aged ≥35 years old and lived in the district for at least 2 years, was selected from each household without replacement. The sampling frame resulted in the selection of 2800 potential participants, of whom 2761 individuals agreed to participant in the investigation and filled out the questionnaire, yielding a response rate of 98.61%. All of the participants are registered residents of the district. Inclusion criteria included: (1) without thyroid diseases, neck masses and deformity, malignant diseases, and secondary hypertension; (2) without pregnancy, lactation and weight control; (3) Han nationality. Finally, a total of 2631 individuals met these criteria and were included in the present analysis. The study was in accordance with the World Medical Association Declaration of Helsinki-Ethical Principles for Medical Research Involving Human Subjects and was approved by the Ethics Committee of China Medical University. A written informed consent form was obtained from all participants after they had been informed of the objectives, benefits and confidentiality of personal information.

### Anthropometric measures

The anthropometric indices were measured using standard techniques and equipments with the subjects in light clothing after an overnight fast. BMI was calculated as weight in kilograms divided by the square of height in meters (kg/m^2^). NC was measured at the level of the laryngeal prominence using a flexible tape, with the subjects in the standing position and the head held erect and eyes facing forward. WC was measured at the level of the midpoint between the lower rib margin and the iliac crest. HC was measured at the level of maximal protrusion of the gluteal muscles. WHR was calculated as WC divided by HC.

### BPs measurements and definition

Based on the standardized procedural guidelines^33, 34^, BPs were measured using a standard mercury-column sphygmomanometer after 15 minutes of rest in the sitting position. At the first examination, BPs were measured in both right arm and left arm. When there is a consistent inter-arm difference, the arm with the higher pressure was used. The average of 3 consecutive measurements to the nearest 2 mmHg in the one selected arm was recorded. Participants were advised not to drink alcohol, tea or coffee, smoke and to take exercise for at least 30 minutes before measuring BPs. Essential hypertension was defined as an average SBP ≥ 140 mmHg and/or an average DBP ≥ 90 mmHg, and/or currently receiving treatment for hypertension with antihypertensive medicine.

### Data collection

All participants underwent a self-administered questionnaire delivered by a face-to-face interview. The questionnaire had two parts: the first section elicited baseline information of demographic characteristic; the second section elicited information on tobacco use, alcohol consumption, salt consumption, and physical activity as well as other health-related information. Smoking and drinking status were categorized as current-, former- and non-smoking or drinking, which were detailed in our previous paper^35^. Physical activity levels were categorized as high (≥3 days of vigorous-intensity activity achieving ≥1500 metabolic equivalent of energy (MET)-minutes/week, or ≥7 days of any combination of walking, moderate- or vigorous intensity activities achieving ≥3000 MET-minutes/week), moderate (not meeting the criteria for the “high” category and ≥3 days of vigorous-intensity activity of at least 20 minutes/day or ≥5 days of moderate-intensity activity or walking of at least 30 minutes/day or ≥5 days of any combination of walking, moderate- or vigorous intensity activities achieving ≥600 MET-minutes/week), and low (not meeting any of the above mentioned criteria) categories^36^.

All participants were asked for permission to collect a blood sample after an overnight fast of >8 hours. The concentrations of TC, TG, HDL-C, LDL-C, and FBG in samples were determined using a Mindray Autoanalyzer (BS 380 type; Mindray Ltd.; Shenzhen, China) in local community health service centers as well as environment and non-communicable diseases research centers, China Medical University. All assays were performed according to the manufacturer’s instructions.

### Statistical analysis

As human body shape differs according to gender, the data for females and males were treated separately. All of the continuous variables were tested for normality by Shapiro-Wilks W test before Student’s t-test, and those non-normally distributed variables were log-transformed to reach normality and variance homogeneity. Differences in the distribution of baseline characteristics between males and females were tested using student’s t-test for continuous variables (age, BMI, WC, HC, NC, WHR, SBP, DBP, FBG, TG, TC, LDL-C, HDL-C and salt consumption) and chi-square test for categorical variables (family history of hypertension, education levels, smoking and drinking status and physical activity). Generalized linear regression models were used to examine the associations of NC with SBP and DBP. Univariate and multivariate logistic regression models were used to assess the association between NC and hypertension risk. We performed univariate regression analysis to evaluate the potential risk factors of arterial BPs and hypertension, and those with *p* < 0.2 in the univariate analysis were included in the multivariate model. Additionally, to avoid the problem of multi-collinearity, BMI and WC were included in the models as categorical variables, because multicollinearity between categorical variables was thought to be not as strong as that between continuous variables^37^. Normal weight was defined as a BMI < 24, overweight was defined as 24 ≤ BMI < 28, and obesity was defined as a BMI ≥ 28^38^. Abdominal obesity was defined based on WC (WC ≥ 85 cm for males and ≥80 cm for females). Therefore, model 1 included only NC. In model 2, we adjusted for age, FBG, salt consumption, smoking and drinking status, physical activity and family history of hypertension. In model 3, we adjusted for the covariates in model 2 as well as BMI categories. In model 4, we adjusted for the covariates in model 2 as well as WC categories. In model 5, we adjusted for the covariates in model 2 as well as WHR. Subsequently, ROC analysis was performed to assess the accuracy of NC as diagnostic test for hypertension, and determine optimal sex, age and BMI-specific cut-offs of NC for predicting hypertension. Logistic regression models were also used to assess the associations of NC (per unit increases of NC) and NC categories (≥cut off value versus < cut-off value) with hypertension risk. All statistical analyses were performed using SPSS software (version 17.0; SPSS Inc,. Chicago, IL, USA). A two tailed *p* value < 0.05 was taken as statistically significant.

## Electronic supplementary material

Supplementary Tables
