# Supplementary material for: Neck circumference associated with arterial blood pressures and hypertension: A cross-sectional community-based study in northern Han Chinese
Source: Sci Rep. 2017 Jun 1;7:2620. doi: 10.1038/s41598-017-02879-7 (PMC5454030; doi:10.1038/s41598-017-02879-7)
Supplement: Supplementary file 1 — Supplementary Tables [file 41598_2017_2879_MOESM1_ESM.doc]

**Neck circumference associated with arterial blood pressures and hypertension: A cross-sectional community-based study in northern Han Chinese**

Shujun Fan^1,†^, Boyi Yang^2,†^, Xueyuan Zhi^1^, Jing He^3^, Ping Ma^3^, Luyang Yu^3^, Quanmei Zheng^1^ & Guifan Sun^1,*^

^1^ Research Center of Environment and Non-Communicable Disease, School of Public Health, China Medical University, Shenyang 110122, China.

^2^ Guangzhou Key Laboratory of Environmental Pollution and Health Risk Assessment, Department of Preventive Medicine, School of Public Health, Sun Yat-sen University, Guangzhou, China.

^3^ Department of Non-Communicable Disease, Shenhe Center for Disease Control and Prevention, Shenyang, Liaoning, China.

^†^ The two authors contributed equally to this work.

**Supplementary Tables**

**Table S1. Univariate linear regression analysis of potential risk factors with arterial blood pressure levels.**

|  | **Systolic blood pressure (mmHg)** | | | **Diastolic blood pressure (mmHg)** | | |
| --- | --- | --- | --- | --- | --- | --- |
| **Variables** | **Estimate** | **95% CI** | ***p_1_*** | **Estimate** | **95% CI** | ***p_2_*** |
| Age (years) | 0.23 | 0.18 – 0.28 | < 0.001 | 0.05 | 0.01 – 0.08 | 0.005 |
| Gender | 3.72 | 2.61 – 4.83 | < 0.001 | 3.36 | 2.63 – 4.08 | < 0.001 |
| Body mass index (kg/m^2^) | 0.78 | 0.64 – 0.92 | < 0.001 | 0.52 | 0.43 – 0.62 | < 0.001 |
| Waist circumference (cm) | 0.40 | 0.35 – 0.46 | < 0.001 | 0.22 | 0.18 – 0.26 | < 0.001 |
| Waist-to-hip ratio | 22.80 | 15.49 – 30.10 | < 0.001 | 9.84 | 5.02 – 14.66 | < 0.001 |
| Fasting blood glucose (mmol/L) | 0.34 | 0.13 – 0.54 | 0.001 | 0.01 | -0.12 – 0.14 | 0.867 |
| Salt consumption (grams/day) | 0.07 | -0.003 – 0.15 | 0.061 | 0.001 | -0.05 – 0.05 | 0.969 |
| Family history of hypertension | 1.40 | 0.27 – 2.53 | 0.015 | 1.71 | 0.97 – 2.45 | < 0.001 |
| Smoking status | 1.23 | 0.48 – 1.99 | 0.001 | 1.41 | 0.92 – 1.91 | < 0.001 |
| Drinking status | -0.02 | -0.62 – 0.58 | 0.950 | 0.93 | 0.53 – 1.32 | < 0.001 |
| Physical activity | -2.73 | -3.87 – -1.59 | < 0.001 | -0.50 | -1.26 – 0.25 | 0.190 |
| History of hypertension | 12.87 | 11.87 – 13.86 | < 0.001 | 6.27 | 5.58 – 6.96 | < 0.001 |

CI, confidence interval.

*p* - value < 0.05 was considered statistically significant.

**Table S2. Univariate logistic regression analysis of potential risk factors with hypertension.**

| **Variables** | **OR** | **95% CI** | ***p*** |
| --- | --- | --- | --- |
| Age (years) | 1.03 | 1.02 – 1.03 | < 0.001 |
| Gender | 1.31 | 1.11 – 1.54 | 0.001 |
| Body mass index (kg/m^2^) | 1.12 | 1.09 – 1.14 | < 0.001 |
| Waist circumference (cm) | 1.05 | 1.04 – 1.06 | < 0.001 |
| Waist-to-hip ratio | 6.64 | 2.21 – 19.97 | 0.001 |
| Fasting blood glucose (mmol/L) | 1.06 | 1.02 – 1.10 | 0.006 |
| Salt consumption (grams/day) | 1.01 | 1.00 – 1.02 | 0.113 |
| Family history of hypertension | 2.11 | 1.78 – 2.49 | < 0.001 |
| Smoking status |  |  | 0.256 |
| Former-smokers | 1.44 | 0.93 – 2.24 | 0.106 |
| Current-smokers | 0.98 | 0.78 – 1.22 | 0.830 |
| Drinking status |  |  | 0.053 |
| Former-drinkers | 2.04 | 1.12 – 3.73 | 0.021 |
| Current-drinkers | 0.95 | 0.80 – 1.14 | 0.592 |
| Physical activity | 0.82 | 0.69 – 0.97 | 0.019 |

OR, odds ratio; CI, confidence interval.

*p* - value < 0.05 was considered statistically significant.
